# Mental health and addiction services and reform in Aotearoa-New Zealand

**DOI:** 10.1192/bji.2025.15

**Published:** 2025-08

**Authors:** Peter McGeorge, Susanna Every-Palmer

**Affiliations:** 1 University of Canberra, Australia; 2 Department of Psychological Medicine, University of Otago Wellington, New Zealand

**Keywords:** Mental health, New Zealand, health policy, substance-related disorders, healthcare reform

## Abstract

This paper reviews the development of Aotearoa-New Zealand’s (New Zealand’s) specialist Mental Health and Addiction Services (MH&A Services) and Mental Health and Addiction plans (MH&A Plans) to improve the mental well-being of all New Zealanders. It does so in the context of New Zealand’s 2022 health reforms, and the government’s response to its 2018 Inquiry into MH&A (He Ara Oranga). First, the context for reform is described, including New Zealand’s sociodemography, existing data monitoring systems, mental health epidemiology and a current overview of its specialist MH&A Services. The findings of the He Ara Oranga Inquiry and the goals of the MH&A plans are then outlined, along with progress in establishing new initiatives related to them. Finally, challenges to the new direction for New Zealand’s MH&A system are reviewed, with possible strategies to address them and other key implementation challenges involving the separation and operationalisation of the main strategies of MH&A plans. A high-level view of MH&A Services is taken, rather than one detailing speciality services such as age-related, cultural or addiction services.

This paper explores the evolution of Aotearoa-New Zealand’s (New Zealand’s)^a^ Mental Health and Addiction (MH&A) services and current policy aimed at enhancing the mental well-being of all New Zealanders. It is set against the backdrop of New Zealand’s 2022 health reforms and the overnment’s response to an inquiry into MH&A (He Ara Oranga) undertaken in 2018 (the Inquiry).^1,2^

In an earlier 2008 paper, progress with New Zealand’s MH&A services was highlighted, noting the shift from institutional to community-based care.^3^ At the time, 3% of the population was estimated to have severe mental conditions, but only 1.5% had accessed MH&A services. Since then, access to MH&A district-based services has been established nationwide, with 67% of care delivered in community settings and overall access to these rising to 3.4%.^4^ Nevertheless, despite funding increasing from NZ$866.6 million in 1994 to NZ$1.45 billion in 2018, the MH&A Inquiry identified major shortcomings in the service availability, quality and inequity of services for Indigenous Māori people. To address these issues, new MH&A plans – Kia Manawanui-Aotearoa^5,6^ and Oranga Hinengaro^7^ – were released by the government based on the Inquiry’s recommendations. These offer dual strategies towards improvement in MH&A in New Zealand: the promotion of mental well-being/prevention of mental distress (the Mental Well-being Programme (MWBP)) and strengthening of existing MH&A services.^b^

## Sociodemography

New Zealand is a South Pacific, British Commonwealth nation with a resident population of approximately 5.3 million. The population is comprised of 18.5% children and adolescents, 64.9% adults (18–65 years of age) and 16.5% older adults. There are slightly more women than men, with a median age of 36.4 years. Ethnically, the population is diverse (in the following data, people were able to identify with more than one ethnicity): 67.3% identify as European, 17.8% as Māori, 8.9% as Pacific peoples, 17.3% as Asian and 1.9% from other backgrounds (e.g. Middle East, South America, Africa).^8^ In the September 2024 quarter, unemployment was 4.8%, inflation was within target at 2.2% (compared with 5.6% in 2023) and household living costs at 3.8% had decreased from 8.2% in December 2023.^9^

The Treaty of Waitangi (Te Tiriti o Waitangi), signed in 1840, remains central to New Zealand society and its healthcare framework. Despite differences in interpretation between the Māori and English versions, widely recognised principles include partnership between the Crown and Māori (whakahoa), cultural protection (whakamaru), participation of Māori in society (pātuitanga), equity (mana taurite) and self-determination (tino rangatiratanga). Addressing health disparities between Māori and non-Māori, by expanding Kaupapa Māori (by Māori/for Māori) MH&A services and ensuring their equity with general services, remains a priority in regard to mitigating the ongoing historical impacts of colonisation.^10^

## National health reforms and governance

Until 2021, New Zealand health services were managed by 20 District Health Boards (DHBs) under the Ministry of Health. In July 2022, the Government disbanded these DHBs, implementing a centralised structure to address fragmentation and disparities in outcomes.

Three national entities were formed:

- The Ministry of Health, MOH (Manatū Hauora), is responsible for health policy, strategy and regulation.
- Health New Zealand (Te Whatu Ora) manages the planning, commissioning and operations of services nationally and regionally.
- The Māori Health Authority (Te Aka Whai Ora) was created to address Māori health disparities but was disestablished in early 2024, with responsibilities integrated into Health New Zealand (HealthNZ), and a focus on Māori representation at regional and district levels.

HealthNZ currently operates under a commissioner, who reports to the Minister of Health. A CEO and four Deputy CEOs manage Public District Health and Speciality Services (HSS), including Public Secondary MH&A Services (PSMHA services) and contracts with non-governmental organisations (NGOs) and, over time, will participate in the establishment and governance of MH&A Integrated Locality Networks.

## MH&A databases, performance and quality monitoring

Monitoring systems for MH&A services include databases and outcome indicator frameworks developed by the Ministry of Health (Manatu Hauora), the Mental Health and Wellbeing Commission (Te Hiringa Mahara, MHWC) and the Health, Quality and Safety Commission (Te Tāhū Whaiora, HQSC). The Programme for Integrated Mental Health Data (PRIMHD) is the primary MH&A database, collecting data on service utilisation, diagnosis, key performance indicators (KPIs), quality and outcomes.^11,12^ Managed by HealthNZ, with the help of Te Pou (a national workforce centre for adult MH&A), PRIMHD is accessible to Crown entities, NGOs, academic institutions and the public. Additional data are sourced from the National Minimum Dataset and the Health Workforce Information Programme.

**Fig. 1:**
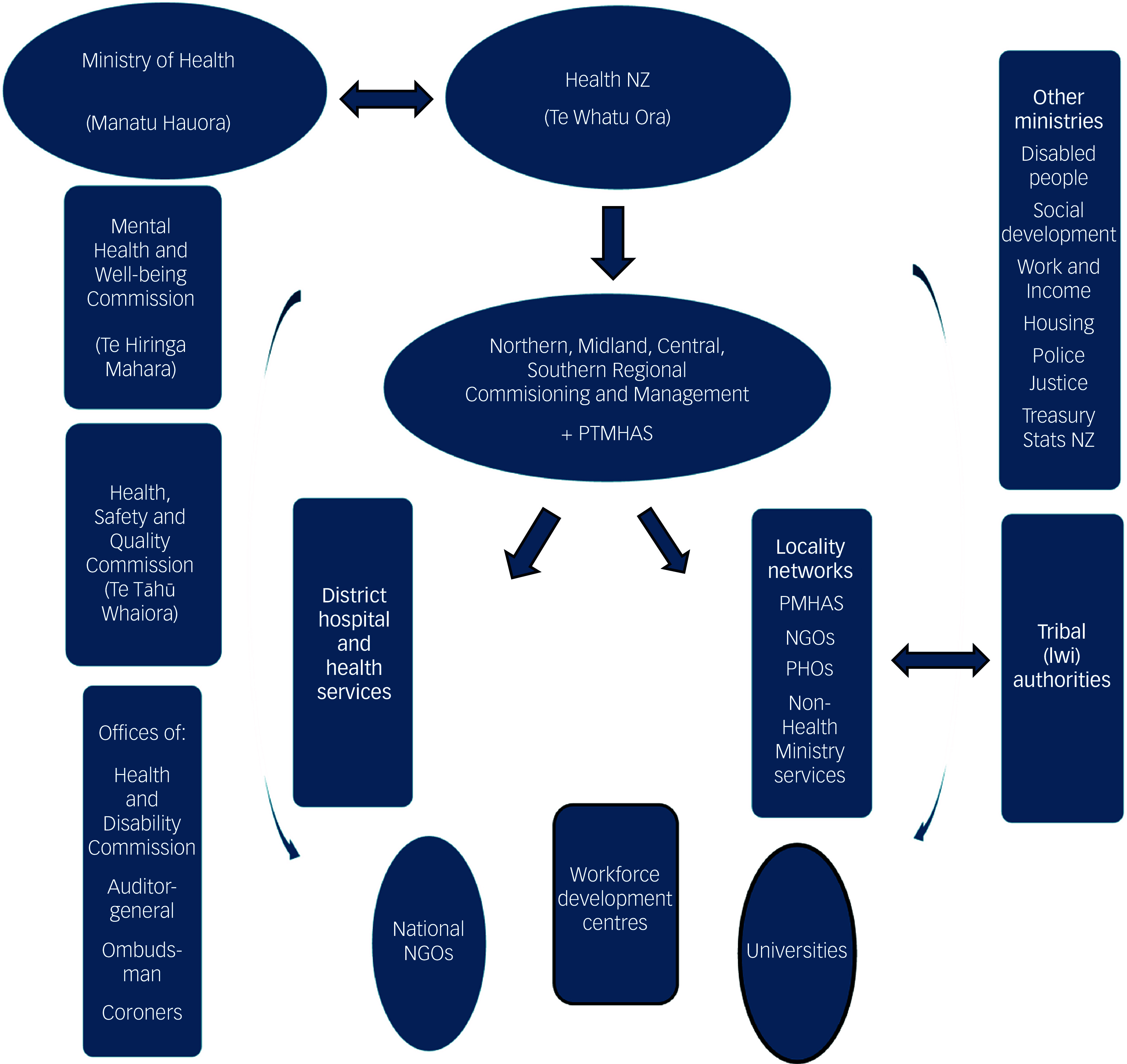
Structure and governance of New Zealand’s Public Mental Health and Addiction Services. NGOs, non-governmental organisations; PHOs, public health organisations; PTMHAS, Public Tertiary Mental Health and Addiction Services.

Although HealthNZ is primarily responsible for commissioning, service management and quality improvement of MH&A services, other agencies, including Ministries, Crown Entities (particularly MHWC and HQSC) and NGOs, play important roles in monitoring and enhancing services (Fig. 1).

Regular surveys conducted by the Ministry of Health (the New Zealand Health Survey, New ZealandHS), Statistics New Zealand and the Treasury provide information on health, mental distress and well-being. These data inform the MHWC and HQSC frameworks for improving safety, quality of care and inter-service transitions. NGOs, such as the national MH&A workforce development centres for adults (Te Pou), children and adolescents (Te Whāraurau), Māori (Te Rau Matatini) and Pacific Islanders (Le Va), contribute to workforce monitoring and training. Te Pou also supports general data and KPI analysis for HealthNZ, while universities play an important role in programme evaluation and service critique.

## Prevalence, access and demand

A comprehensive national prevalence survey of mental disorders has not been conducted since 2003–2004 (Te Rau Hinengaro), limiting the understanding of current and future MH&A needs.^13^ However, a review of data from Te Rau Hinengaro and New ZealandHS data by the University of Otago for the MH&A Inquiry indicates that the prevalence of disorders such as depression, anxiety, substance use disorders and common psychotic disorders is high in New Zealand and comparable with that in Australia, Europe and the USA.^12^

According to the 2023–2024 New ZealandHS data, 13% of people reported high or very high levels of psychological distress, an increase of 4.7% since 2018–19 and 9.4% since 2012. Distress was significantly higher among Māori, disabled, socioeconomically disadvantaged and youth, ranging from 20 to 30%.^14^

The national suicide rate for 2023–24 of 11.2 per 100 000 was 3.6% lower than the average rate for the past 15 years; however, while trending in the right direction, this reduction did not achieve statistical significance. Māori suicide rates remain disproportionately high, at 16.3 per 100 000, while those for Pacific Island and Asian people are below general population rates.^15^

**Fig. 2:**
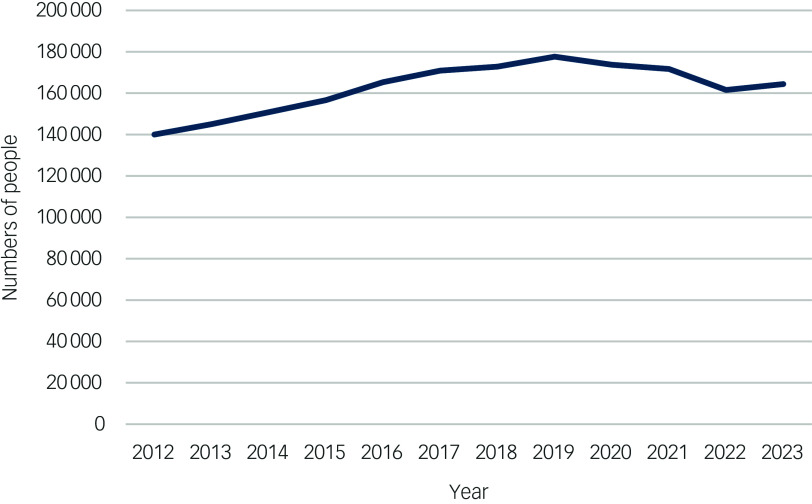
Access to community Public Secondary Mental Health and Addiction Services and non-governmental organisation services, 2012–2023.

Data extracted by Te Pou from PRIMHD show that access to specialist community MH&A services increased steadily from 2012 to 2019 (Fig. 2), followed by a decline during, and stabilisation following, COVID-19.^11^ In-patient admissions, including acute, rehabilitation and forensic services, showed a similar pattern, although the drop in admissions during COVID was more marked for acute in-patients. Trends need to be evaluated, but may reflect a mix of increasing severe mental distress, barriers to residential care associated with staff vacancies, increased clinical complexity during COVID-19 and new community services.

## Structure and capacity

Current MH&A plans advocate for increased focus on integrated locality networks. However, most services are still managed by district-based urban centres, with a notable divide between larger urban and smaller urban/rural areas. Larger urban areas generally benefit from a more extensive range of services, while semirural and rural areas struggle with more limited resources.

**Fig. 3:**
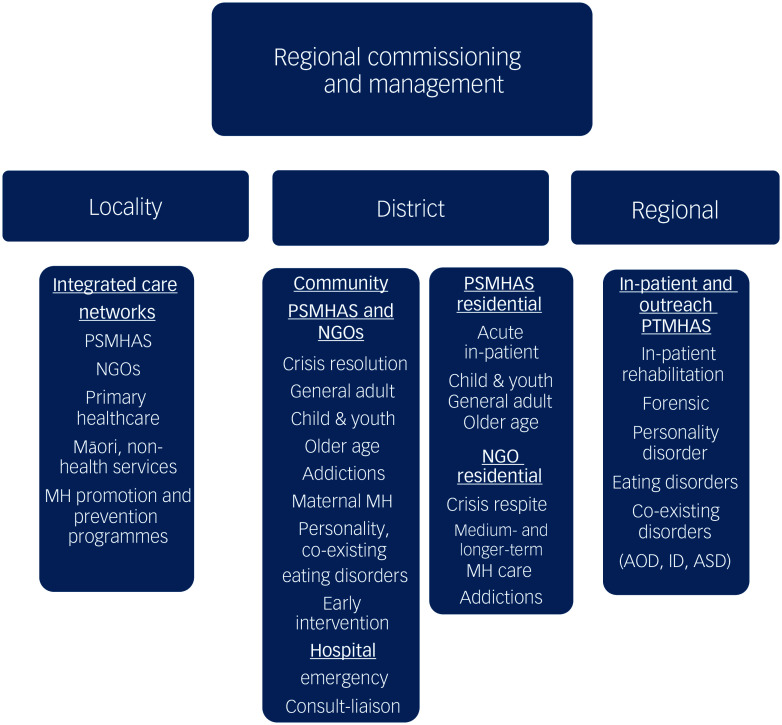
Regional, district and locality configuration of New Zealand’s Mental Health and Addiction Services. NGO, non-governmental organisation, PSMHAS, Public Secondary Mental Health and Addiction Services; PTMHAS, Public Tertiary Mental Health and Addiction Services; MH, mental health; AOD, alcohol and other drugs; ID, intellectual disability; ASD, autistic spectrum disorder.

District services include configurations of pan-ethnic, age-related MH&A services for the general population, specific services for Māori (Kaupapa Māori), Pacific Island and Asian people and addiction and subspecialty services. Emerging locality-based services are supported by district services. In turn, district services are linked to regional tertiary services. Social support services are offered by other government departments such as the Ministries for Social Development, Disability and Children (Fig. 3). In addition to face-to-face services, online therapy options – including websites, digital tools and online telehealth services – have been established and are accessed by 9.5% of the population.^4^

A stocktake of facilities and services conducted by HealthNZ in 2024 has not yet been published. Although PRIMHD has data for in-patient services and community residential care, data for non-residential services largely refer to service utilisation and workforce, rather than measures of capacity and occupancy. Although the situation should be corrected by the new stocktake, this discrepancy limits a fuller assessment of the system’s community capacity and comparisons with international benchmarks.

In-patient data, however, show that New Zealand has 33 mental health beds per 100 000 people, significantly fewer than the European average of 67 beds per 100 000. Of these in New Zealand, there are 12 acute beds per 100 000. Although an undue focus on bed numbers has been criticised,^16^ the New Zealand situation and other MH&A service shortfalls – such as poorly integrated care and insufficient NGO residential services – contribute to delays in the transfer of patients from emergency departments, high bed occupancy rates (often exceeding 95%), high seclusion rates, increased average length of stay (currently 19 days) and ongoing 30% backlogs of people in acute units waiting for step-down residential care.^17^ Children, adolescents and older adults face similar demands, although detailed data are less accessible than those for adult services. Māori and Pacific peoples are disproportionately subjected to involuntary care under the Mental Health Act and seclusion, highlighting inequities that need urgent attention.^18^

Financially, the burden of MH&A care is estimated at NZ$12 billion, accounting for 5% of New Zealand’s gross domestic product. Operational funding for specialist and primary MH&A services now stands at NZ$1.99 billion, which is a major increase from earlier decades. However, funding distribution discrepancies exist. General adult MH&A services, which serve 64.9% of the population, receive 61.8% of funding and are the dominant service provider (personal communication, HealthNZ). They provide support for infant, child and youth and older person services where there are shortfalls in smaller districts and localities.

**Fig. 4:**
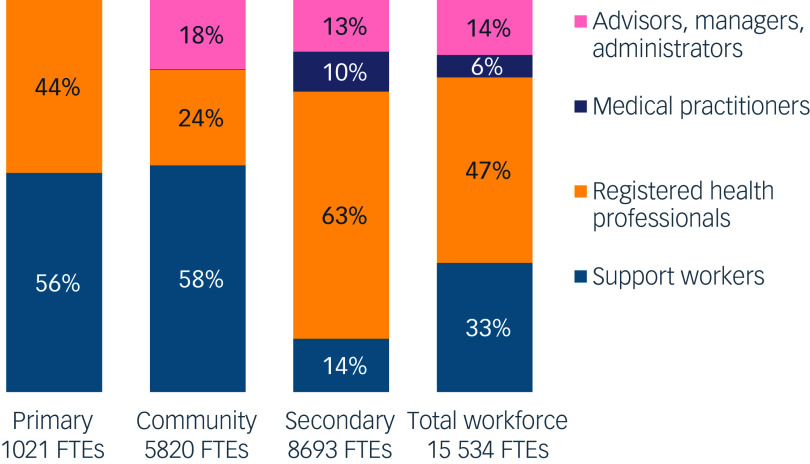
Estimated full-time equivalent (FTE) workforce (employed and vacant) composition, by healthcare setting and role groups, 2022.^19^ Note: support workers are employed in various capacities, including lived experience and Maori and Pasifika cultural roles, health coaching and others not requiring professional registration.

The MH&A workforce has doubled since 2008, driven by increased funding and improved workforce tracking through the National Health Workforce Information Programme. The contributions of NGOs, community support, peer workers and Māori and Pacific staff are now more clearly defined, demonstrating the diversity of roles within the system. Despite this growth, significant issues remain (Fig. 4). Vacancy rates stand at 11.1% overall, with higher rates among some disciplines including psychiatry (20%) already having low numbers (13.6 per 100 000 compared with an average of 18.0 in Organisation for Economic Co-operation and Development countries).^20^

Although a national workforce plan needs to be urgently updated, the background to these issues is complex and includes pay parity issues among secondary MH&A services, NGOs and other government sectors, as well as the migration of MH&A staff to new services in primary healthcare organisations and overseas. The skill sets, work roles, interdisciplinary and interagency functioning required for the new system, are, however, likely to pose even greater challenges. For example, the dilution of roles for which people have been trained, and how multidisciplinary teams are expected to function in terms of leadership and responsibility, may exacerbate problems with recruitment rather than alleviate them.

## Planning and development

The 2018 MH&A Inquiry identified critical issues affecting New Zealand’s mental health services, including insufficient consideration of social and economic determinants of MH&A, support for the identity and equity of Māori and Pacific peoples, undue public access to alcohol, school bullying, youth suicide and the negative impact of social media on mental health. The Inquiry also raised concerns about poor access to specialist care, poorly integrated, overly medicalised and coercive care, outdated mental health legislation, the poor standard of MH&A facilities and limited therapeutic options, such as ‘talking therapies’. The Inquiry made 40 recommendations, 38 of which were fully accepted or accepted in principle by the government. The two that were excluded involved establishing a new social well-being agency and setting a target 20% reduction in suicide rates by 2030.^21^

**Fig. 5:**
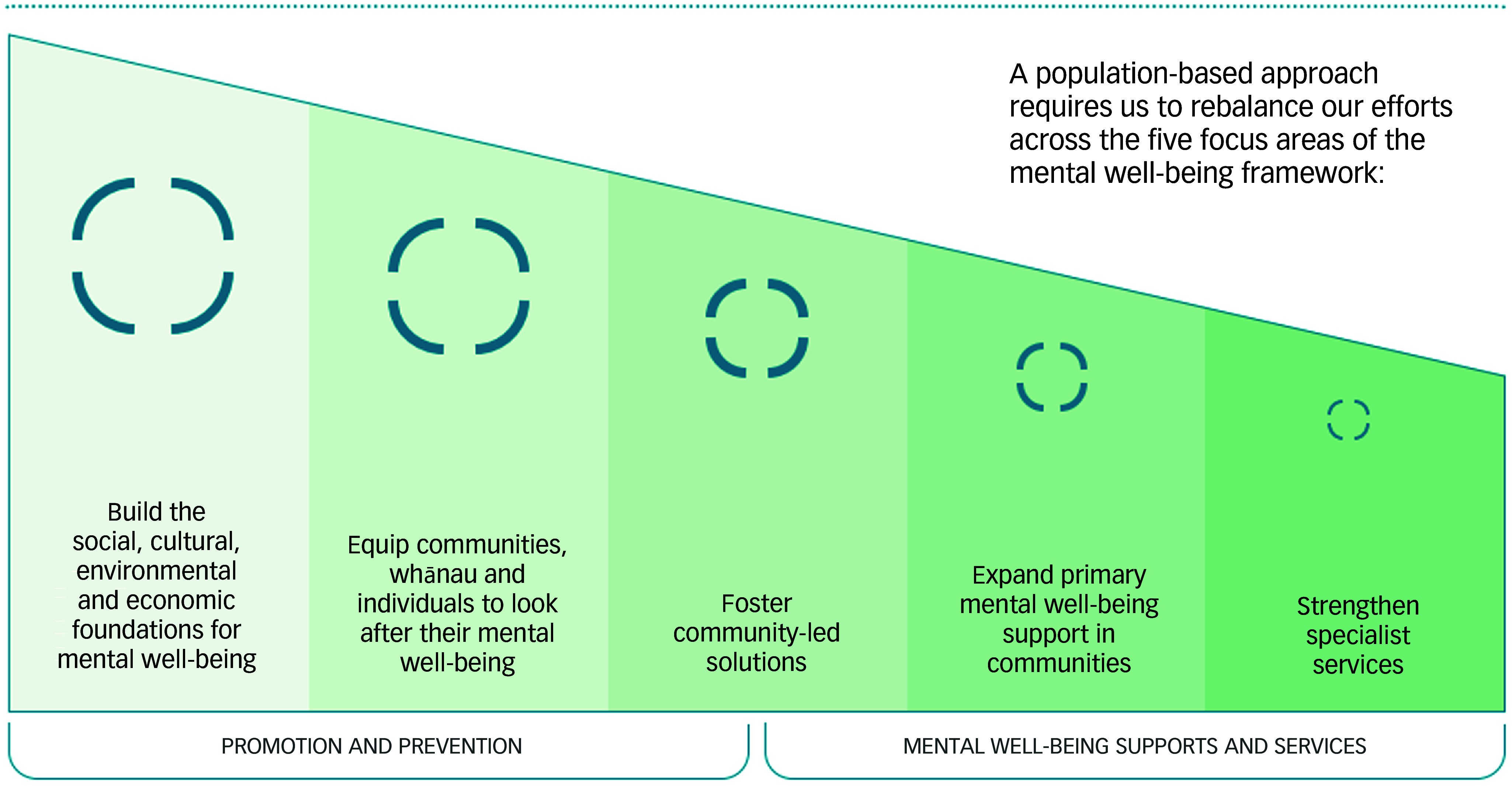
Kia Manawanui-Aotearoa goals.^6^

Kia Manawanui-Aotearoa represents the government’s initial response to these recommendations. Guided by the vision of the Pae Ora (Healthy Futures) Act 2022, it focuses on five goals grouped under two key strategies: promotion and prevention (MWBP) and mental well-being supports and services (MH&A Services) (Fig. 5).

An update to Kia Manawanui was published in 2023.^7^ This details progress made with inter-ministry and regional strategies in addressing the social determinants of mental well-being through the funding of leadership and support positions. A new primary healthcare ‘access and choice’ programme for people, building on the work of recent decades,^22^ has been implemented across the country for people with mild to moderate mental distress, with reports of high usage (3.7% of the population) and patient satisfaction.^23^

To strengthen MH&A services, a ‘system and service’ framework (Oranga Hinengaro) is being implemented and an expanded array of crisis resolution, Kaupapa Māori (by Māori/for Māori) and youth services funded.^6^ Some in-patient units are being refurbished or replaced, although it is not clear whether more beds will be added to the total bed numbers.^24^ The Mental Health Act (1992) is also being repealed and replaced to better align with human rights, supported decision-making and a recovery and well-being approach.^25^ These changes are aimed at reducing compulsory and coercive treatment, upholding the principles of the Treaty (Te Tiriti o Waitangi) and the International Convention on the Rights of Persons with Disabilities and involving peers and family in decision-making. Less emphasis on terminology relating to mental disorders and risk management in the assessment processes, and more on mental capacity, has also been signalled.^22,26^

## Critiques and challenges

Despite these initiatives, critiques remain. Allison et al, for example, raised concerns about whether the approach advocated by the MH&A Inquiry to establish a ‘Big Community’ system, as opposed to the previously described “Big Psychiatry’ model, would disadvantage people with more severe MH&A disorders and have little benefit in reducing suicide rates and demand for acute crisis and in-patient services. They argued that New Zealand’s MH&A services, compared with international benchmarks (see above), actually resembled a ‘Small Psychiatry’ system.^2^ Moreover, Mulder et al, from the same group, questioned the direction of MH&A Inquiry recommendations, which called for investment in psychotherapeutic options, saying that thedr would be used primarily by ‘middle-class’ patients and that similar programmes had failed in Australia and the UK. They advocated for better funding of MH&A services, especially in socially deprived areas where the numbers of people with severe disorders and suicides are higher.^25,27^

A survey conducted by Every-Palmer and her colleagues indicated that New Zealand psychiatrists felt that the MH&A sector was ‘not fit for purpose’ and ‘heading in the wrong direction’, as evidenced by increasing workloads, after-hours demand, case complexity and workforce vacancies, and by poor service access and fragmented care.^28^ Again, the Association of Salaried Medical Specialists, while seeing the move towards greater mental health promotion and prevention as ‘positive’, cast doubt on whether the expectation that the measures recommended by the MH&A Inquiry ‘will relieve pressures on specialist services in the “short or medium terms”’. Their report identified service shortages and staff vacancies and called for funding for serious MH&A difficulties to be extended to 5% of the population rather than the current level of 3%.^17^

The concerns of these authors reflect the MH&A Inquiry findings regarding the state of MH&A services. However, they prioritise improvement in MH&A services, rather than emphasising the central importance of the MWBP, as appears to be the case in the MH&A planning documents. These deserve serious attention, especially if expanded psychological therapies for people with mild-to-moderate disorders, psychosocial community interventions and population-based well-being strategies do not improve the existing pressures on MH&A services. Moreover, while the broader vision of mental health and well-being has received widespread support in New Zealand,^29^ there are operational matters that are not well addressed in the MH&A plans. These relate not only to the direction of the plans but also to the merging of MHWP with MH&A services, which has implications for the funding, workforce, implementation processes and operational systems required for the successful functioning of their respective programmes.

## Discussion

The development of pathways to mental well-being for all New Zealanders, and an effective system of integrated care and treatment for people facing significant mental distress and disability, is a worthy albeit complex endeavour. The current plans and services provide a substantial foundation for achieving the dual objectives specified by the former. They outline the initiatives, outcomes to be achieved and the systems they are intended to leverage. However, their success is contingent upon adequate funding, well-staffed expert services, changes in work roles/team functioning and political and management systems aligned with clearly stated objectives.

Adequate funding is fundamental for dual strategies in the planned MH&A system. To achieve this, funding for MH&A services should be increased to address the needs of the 5% of the population with serious conditions, as advocated by professional bodies. MWBP should also be costed separately and realistically funded, rather than drawing on the already limited resources of existing MH&A services.

Support for a national workforce plan is a high priority. In this, the recruitment and training of MH&A staff to work with people from diverse community and cultural backgrounds and other organisations is essential. Furthermore, separate recruitment and management of public health specialists is important for the MH&A sector in achieving its objectives.

To facilitate these ends, we suggest that the MWBP and MH&A service arms of the system be managed separately. A dual management approach would not only clarify the distinct objectives of each programme, but would enable the coordination of responsibilities where they overlap, and their integration with other health and social services.

Apart from detailed modelling of funding and workforce requirements, regions need up-to-date ecosystem information to prioritise planning and ensure that sufficient capacity or alternatives are available to meet their goals. Communities experiencing social adversity and, where it exists, discrimination, should be prioritised and engaged in the development of MH&A plans and the co-production and local monitoring of service provision wherever possible. Such co-production must include both people with lived experience and Māori.

At the service delivery level, key challenges include implementing agreed, evidence-based models of integrated care tailored to New Zealand’s cultural and demographic realities. Although DHB models exist, these should be aligned with international standards for integrated care^30^ and monitored consistently across localities using MHWC, HQSC and international quality frameworks for fidelity and quality. In this regard, the major bodies involved in the governance, monitoring and support of MH&A services and well-being should themselves be rationalised and better integrated than is currently the case.

An updated national prevalence study of mental disorders is urgently required, and should be undertaken within the next 2 years. Ongoing, long-range research and evaluation of MWBP and MH&A services, based on validated health ecosystem and implementation science approaches, such as Salvador-Carulla’s DESDE-LTS service mapping tool and Beer’s viable system model, are also critical to both the effective implementation of the new system and meeting the scrutiny of a future Inquiry.^31,32^

In conclusion, New Zealand’s MH&A strategy is an ambitious but crucial undertaking. With operational clarification of its implementation and ongoing evaluation, New Zealand’s approach to promoting mental well-being and improving mental health and addiction care in a culturally diverse population could serve as a model for other nations experiencing similar challenges.

## Dedication

This publication is dedicated to Dr Peter McGeorge, OBE, the first author and a mental health leader in New Zealand and Australia for many decades. Peter completed this paper while he was unwell. He passed away peacefully in hospital on 27 March 2025 just before it was published. *Kua hinga te totara i te wao nui a Tane*(a great totara tree has fallen in Tane’s forest).

## Footnotes

a. New Zealand is a multi-cultural nation founded on a bicultural basis, with English and indigenous Māori as its official languages. For international readers, where dual languages are referenced, English is generally used first followed initially by the Māori version in brackets then English is used consistently. Where Mori is usually referenced in New Zealand, Māori is used first followed initially by English in brackets then Māori is used consistently.

b. Kia Manawanui refers to these two strategies as Promotion and Prevention, and Mental Well-being Support and Services, the latter of which cover Primary Healthcare MH&A services and specialist MH&A services. MH&A Services are used in the paper rather than Mental Well-being Support and Services to make a distinction from the Mental Well-being Programme and its acronym (MWBP).
